# The impact of Thai multidisciplinary smoking cessation program on clinical outcomes: A multicentre prospective observational study

**DOI:** 10.3389/fpubh.2022.965020

**Published:** 2022-08-24

**Authors:** Chayutthaphong Chaisai, Kednapa Thavorn, Somkiat Wattanasirichaigoon, Suthat Rungruanghiranya, Araya Thongphiew, Piyameth Dilokthornsakul, Shaun Wen Huey Lee, Nathorn Chaiyakunapruk

**Affiliations:** ^1^School of Pharmacy, Monash University Malaysia, Bandar Sunway, Selangor, Malaysia; ^2^Department of Pharmaceutical Care, Faculty of Pharmacy, Chiang Mai University, Chiang Mai, Thailand; ^3^School of Epidemiology and Public Health, University of Ottawa, Ottawa, ON, Canada; ^4^Ottawa Hospital Research Institute, Ottawa, ON, Canada; ^5^Thai Physician Alliance Against Tobacco, Bangkok, Thailand; ^6^Faculty of Medicine, Srinakharinwirot University Ongkarak, Bangkok, Thailand; ^7^Paolo Phaholyothin Hospital (BDMS), Bangkok, Thailand; ^8^Center for Medical and Health Technology Assessment (CM-HTA), Department of Pharmaceutical Care, Faculty of Pharmacy, Chiang Mai University, Chiang Mai, Thailand; ^9^School of Pharmacy, Taylor's University, Bandar Sunway, Selangor, Malaysia; ^10^Department of Pharmacotherapy, College of Pharmacy, The University of Utah, Salt Lake City, UT, United States; ^11^IDEAS Center, Veterans Affairs Salt Lake City Healthcare System, Salt Lake City, UT, United States

**Keywords:** smoking, smoking cessation, tobacco, program evaluation, health policy

## Abstract

**Introduction:**

Tobacco use is the leading preventable cause of morbidity and mortality worldwide. Since 2010, Thailand has implemented a multidisciplinary smoking cessation clinic, which provides smoking cessation services, but the effectiveness of the clinics was not formally evaluated. This study was conducted to assess the real-world effectiveness of this multidisciplinary smoking cessation program.

**Methods:**

We conducted a prospective, multicentre, observational study on Thai participants aged 13 years and older in 24 smoking cessation clinics across Thailand's 13 health regions. Each clinic offered smoking cessation interventions according to the well-established 5As model for smoking cessation (Ask, Advise, Assess, Assist, and Arrange). Outcomes of interest were continuous abstinence rates (CAR) at 3 and 6 months. Biochemical confirmation and self-reporting were used to assess the outcomes. Descriptive statistics (mean, SD, median, IQR, and percentage) were used to analyze the smoking cessation outcomes in both intention-to-treat and per-protocol analysis approaches.

**Results:**

Smokers receiving services from the Thai multidisciplinary smoking cessation clinics had CAR of 17.49 and 8.33% at 3 and 6 months, respectively. For those with cardiovascular disease (CVD) or cerebrovascular disease, CAR was found to be 26.36% at 3 months and 13.81% at 6 months. While participants with chronic obstructive pulmonary disease (COPD) had CAR ranging from 32.69% at 3 months to 17.31% at 6 months.

**Conclusion:**

The multidisciplinary team smoking cessation clinic was effective in assisting smokers in quitting smoking. The effectiveness of the clinic was more pronounced for smokers with CVD, cerebrovascular disease, or COPD. Findings from this study support a decision to include multidisciplinary smoking cessation clinics in the universal health care benefits package.

## Key messages

- Smoking cessation is a global priority and previous studies have shown that smoking cessation programs were beneficial in assisting smokers to quit smoking and reducing the number of smokers.
- Since 2010, Thailand has implemented multidisciplinary smoking cessation clinics, but the efficacy of the clinics was measured based on retrospective self-reported data without biochemical confirmation. This prospective study measured the effectiveness of the clinics using both self-reported and biochemical outcomes.
- Findings from this study demonstrated that the Thai multidisciplinary smoking cessation clinics were effective at assisting individuals to quit smoking and the results could inform the decision to add the multidisciplinary smoking cessation clinics to the universal health care benefits package.

## Introduction

Tobacco use is the leading preventable cause of morbidity and mortality worldwide (1). Tobacco-related mortality accounts for 8.2 million deaths per year, with 7 million deaths occurring among tobacco users and 1.2 million deaths occurring as a result of second-hand smoke exposure. The number of smokers continues to grow; of these, approximately 80% live in low- and middle-income countries (LMIC) (1, 2). In Thailand, the prevalence of smoking among Thai adults aged 15 and older was 19.10% in 2017, according to the latest national survey. However, the prevalence of smokeless tobacco use was not provided because Thai law prohibits the importation and manufacture of smokeless tobacco products. Tobacco use has been shown to link to several non-communicable diseases, including cardiovascular disease (CVD), chronic obstructive pulmonary disease (COPD), cerebrovascular disease (e.g., stroke), and cancer, most notably lung cancer (1–6).

Smoking cessation is a global priority to reduce tobacco-related morbidity and mortality, (1, 2, 5). A systematic review and meta-analysis of randomized controlled trials (7) revealed that smoking cessation programs that relied solely on self-help strategies without providing face-to-face counseling were less effective than those that included face-to-face counseling, whereas programs that included brief advice intervention, defined as providing brief individual advice and information on how to quit smoking, were more effective. Additionally, smoking cessation programs that included behavioral change strategies, such as a series of meetings with smokers, were more effective than usual care and brief advice alone, with a success rate of over 3% to 9% and 4% to 5%, respectively.

Currently, only 26 countries, which account for one-third of the world's population, have national comprehensive cessation services with full or partial cost coverage (1). Thailand has established a multidisciplinary smoking cessation clinic, namely the SMART Quit Clinic Program (FAH-SAI Clinic), since 2010, which provides smoking cessation services through a multidisciplinary team, including education counseling, pharmacological treatment, as well as the prevention, monitoring, and empowerment of smokers. Multidisciplinary smoking cessation clinics currently operate in 552 locations throughout Thailand's 77 provinces. According to the published multidisciplinary smoking cessation clinics' annual report (8), the self-reported point prevalence of smoking cessation at 3, and 6 months was 33.9%, and 38.2%, respectively. However, this report was based on self-reported data without biochemical confirmation. Thus, this study sought to determine the real-world effectiveness of Thai multidisciplinary smoking cessation clinics based on continuous abstinence rates (CAR) at 3 and 6 months through a prospective cohort design with a biochemical validation of smoking status.

## Methods

### Study design

We conducted a multicentre prospective observational study without a control group since multidisciplinary smoking cessation clinics are considered the standard care for smokers in Thailand.

### Study setting

This study focused on 24 multidisciplinary smoking cessation clinics across Thailand's 13 health regions. To ensure that the study samples represent geographical regions nationwide, we used a stratified random sampling based on the following criteria 1) the previous year's recruitment rate and the number of visits to each setting; and 2) multidisciplinary smoking cessation clinics were located using Thailand's 13 health regional strata. Our study sample consisted of individuals receiving smoking cessation services from 2 university hospitals, 10 tertiary hospitals, 11 secondary hospitals, and 1 private hospital. We estimated the required sample size based on a previous report by the Thai multidisciplinary smoking cessation clinics (8), which suggested the success rate for smoking cessation at 6 months of 38.2%. The sample size is calculated using the one group proportion formula at a two-tailed alpha level of 0.05 and an 80% power level. An additional 10% of cases were included to account for attrition. As a result, a minimum of 1,540 participants are required to ensure that we have sufficient statistical power to represent the outcomes in the population.

### Study population

#### Inclusion criteria

We recruited individuals aged 13 years or older who are in the contemplation or action stage and first commence to smoking cessation program in the multidisciplinary smoking cessation clinics.

#### Exclusion criteria

Participants were excluded from the study if they have been diagnosed with any cancers or intellectual disabilities that impair their ability to complete the exhaled carbon monoxide (CO) testing at the time of eligibility screening.

### Description of multidisciplinary smoking cessation clinics

Multidisciplinary smoking cessation clinics involve multidisciplinary health care providers, including physicians, nurses, nursing assistants, pharmacists, dentists, public health officers, psychologists, and practitioners of traditional Thai medicine. The clinics provide cessation services according to the well-established 5As model of smoking cessation (Ask, Advise, Assess, Assist, and Arrange). Specific interventions included 1) identifying, diagnosing, and documenting tobacco use status; 2) assessing the severity of a person's nicotine dependence; 3) counseling the patient to abstain from tobacco use; 4) patients who are willing to give up smoking are evaluated; 5) assisting patients in quitting smoking through counseling or pharmacological interventions; 6) establishing a schedule follow-up contacts. However, interventions and activities might vary slightly across settings, depending on the local context and human resource availability; for example, some settings provide home visits while others provide group counseling.

### Outcome measures

The primary outcome of interest in this study was a continuous abstinence rate (CAR) at 3 and 6 months. We also reported a 7-day self-reported [prevalence abstinence rate (PAR)], and 7-day exhaled CO-confirmed point prevalence abstinence rate (CO-confirmed PAR), as well as smoking cessation outcomes for patients diagnosed with CVD or cerebrovascular disease, and COPD at 3, and 6 months.

This study used two outcome measurement methods (9, 10), including a self-reported questionnaire and a gold standard biochemical validation method, i.e., exhaled CO test. Each method assesses the prevalence of abstinence for 7-day abstinence (PAR) at 3 and 6 months. In comparison, the exhaled CO-confirmed 7-day point prevalence abstinence (CO-confirmed PAR) for exhaled CO methods was defined as exhaled CO concentrations are < 10 parts per million (ppm). All CO-oximeters used in this study were calibrated and validated prior to being used on subjects.

Participants were asked whether they had ever used any form of smoking in the past at two points in time (3 and 6 months). The 7-day point PAR is determined by the follow-up question “Have you used any smoking products (of any type) in the last 7 days?” Those who respond that they have not used smoking products in the last 7 days and have fixed CO concentrations in exhaled air < 10 ppm are considered to have quit at the follow-up visit. The continuous abstinence rate (CAR) is determined by the follow-up questions “When did you quit smoking?” and “Have you used any smoking products (of any type) since you quit?” The quit date will be noted, and those who respond that they have not used smoking products in any form since the quit date are considered to have quit (depending on the duration).

### Statistical analysis

Baseline characteristics of the participants were described using descriptive statistics (mean, SD, median, IQR, and percentage). We analyzed the smoking cessation outcomes using both intention-to-treat and per-protocol analysis approaches. All data were analyzed using STATA version 14.

### Ethics and dissemination

Thailand's Ethical Review Committee for Human Subjects Research has approved the protocol for this study, protocol number 24/2562 and document number 51/2019.

### Patient and public involvement

Patients or the public were not involved in the design, conduction, reporting, or dissemination plans of our research.

## Results

### Sociodemographic, clinical characteristics, and smoking patterns of the participants

A total of 2,041 participants were included in this study. Socio-demographic, clinical, and smoking status of study participants are summarized in Table 1. The participants' mean age was 44.56 years (standard deviations; *SD* = 17.41), and 24.06% of participants were between the ages of 18 and 30 years (24.06%). Most participants were men (90.79%) and married (54.48 %). Most study participants worked in the labor sector, had a high school education, and earned an average monthly personal income of 8,211.36 Thai baht (230.21 US Dollars). Most participants (41.16%) were current-alcohol users, followed by non-users (37.48%). A majority of participants (75.94%) were covered under the universal coverage reimbursement scheme.

**Table 1:** Sociodemographic, clinical characteristics, and smoking pattern of the participants.

**Variables**	***N* = 2,041**	**(%)**
**Age (Mean, SD)**	**44.56**	**17.41**
Less than 18	67	3.28
18–30	491	24.06
31–40	275	13.47
41–50	395	19.35
51–60	354	17.34
More than 60	417	20.43
Not known	42	2.06
**Sex**		
Male	1,853	90.79
**Marital status**		
Married or living with partner	1,112	54.48
**Occupation**		
Unemployed	191	9.36
Private employee	178	8.72
Laborer	1,072	52.52
Farmer	197	9.65
Other	223	10.93
Not Known	180	8.82
**Education**		
≤ High school	1,603	78.54
Vocational college, bachelor or higher	268	13.13
Not known	170	8.33
**Alcohol intake**		
Current user	840	41.16
Ex-user	400	19.60
Non-user	765	37.48
Not known	36	1.76
**Personal income (Bath/Month) (Mean, SD)**	8,211.36	11,239.20
**Personal income (US Dollars/Month) (Mean, SD)**	230.21	315.10
**Personal income (Bath/Month) (Median, p25-p75)**	6,000.00	3,000–10,000
**Personal income (US Dollars /Month) (Median, p25-p75)**	168.21	84.11–280.36
**Reimbursement scheme**		
National health security office: Universal coverage	1,550	75.94
Government	113	5.54
Social security office	257	12.59
Other	64	3.14
Not known	57	2.79
**Comorbidities**		
No comorbidities	1,019	49.93
Hypertension	444	21.75
Cardiovascular diseases or cerebrovascular diseases	243	11.90
Diabetes mellitus	182	8.92
Emphysema or chronic obstructive pulmonary disease	52	2.56
Asthma	48	2.35
Other	385	18.86
Not known	36	1.76
**Number of comorbidities (*****N*** **= 2,005)**		
No comorbidities	1,019	49.93
1	707	34.64
≥ 2	279	13.68
Not known	36	1.76
**Types of smoking**		
Cigarette	1,311	64.23
Hand-rolled, natural, light cigarette	844	41.35
E-cigarette	15	0.73
Not known	36	1.76
Mixed types smoking	180	8.82
**Amount of cigarette per day**		
Average (Mean, SD)	11.82	7.76
Less than 6	544	26.65
6–10	709	34.74
11–20 and higher	752	36.84
Not known	36	1.76
**Time to smoke after waking up**		
Less than 30 min	1,188	58.21
60 min	463	22.68
120 min	329	16.12
Not known	61	2.99
**Exhaled carbon monoxide (ppm) (Mean, SD)**	**7.93**	**5.54**
**Exhaled carbon monoxide (ppm) (Median, p25-p50)**	**7**	**4–11**
**FTND (Mean, SD)**	**4.20**	**2.26**
**FTND (Median, p25-p50)**	**4**	**3–6**
**The severity of nicotine dependence**		
Very low dependence	419	20.53
Low dependence	647	31.70
Medium dependence	263	12.89
High dependence	319	15.63
Very high dependence	172	8.43
Not known	221	10.83

Half of the study participants had no comorbidity (49.93%). Of those with comorbidities, the most common comorbidity was hypertension (21.75%), followed by cardiovascular and cerebrovascular diseases (11.90%), diabetes mellitus (8.92%), and emphysema or chronic obstructive pulmonary disease (2.56%). The most frequently used smoking product was the cigarette (64.23%), followed by hand-rolled, natural, or light cigarettes, and mixed-type smoking accounted for 41.35 and 8.82%, respectively. The average number of cigarettes smoked per day was 11.82 (*SD* = 7.76), with the majority smoking between 11 and more than 20 cigarettes per day. Most study participants started smoking within 30 min after awakening. Our participants were classified as having low dependence, according to exhaled-CO, and the Fagerstrom Test for Nicotine Dependence (FTND) (Table 1).

### Medication and intervention characteristics of the participants

Approximately two-thirds of the study participants (68.40%) received one form of smoking cessation medication (Table 2). We found that nicotine gum was the most commonly used standard monotherapy (80.81%), while a special mouth wash (11) was the most used non-standard monotherapy option (67.5%). Among study participants who received combination therapies, the primary regimen was nortriptyline-based combination therapies (49.77%), followed by nicotine replacement-based combination therapies (45.25%), and bupropion-based combination therapies (4.98%), whereas a special mouthwash in combination with *Vernonia cinerea* (L.) Less was used as the primary in the non-standard combination therapy subgroup (80.95%).

**Table 2:** Medication and intervention characteristics of the participants.

**Variables**	***N* = 2,041**	**(%)**
**Receiving any medication**	**1,396**	**68.40**
**Receiving monotherapy (*****N*** **= 902)**		
**Receiving standard monotherapy (*****N*** **= 99)**		
Nicotine gum	80	80.81
Nortriptyline	16	16.16
Other	3	3.03
**Receiving non-standard monotherapy (*****N*** **= 803)**		
Special mouthwash	542	67.50
Vernonia cinerea (L.) Less (Little ironweed)	186	23.16
Cytisine	75	9.34
**Receiving combination therapies (*****N*** **= 494)**		
**Receiving one of standard combination therapy (*****N*** **= 221)**		
Nortriptyline-based combination regimen	110	49.77
Nicotine-based combination regimen	100	45.25
Bupropion-based combination regimen	11	4.98
**Receiving non-standard combination therapy (*****N*** **= 273)**		
Special mouthwash with Vernonia cinerea (L.) Less	221	80.95
Cytisine-based combination regimen	52	19.05
**Service users**		
Patients without caregiver	1,692	82.90
Patients with caregiver	293	14.36
Not known	56	2.74
Individual counseling	1,671	81.87
Group counseling	128	6.27
Individual counseling and group counseling	181	8.87
Not known	61	2.99
**Types of Intervention**		
Brief advice in hospital/clinic	1,787	87.56
Rehabilitation camp	188	9.21
Other	9	0.44
Not known	57	2.79

Table 2 describes the characteristics of interventions used in this study. The study participants tended to self-refer to multidisciplinary smoking cessation clinics (82.9%) and received individual counseling as a primary intervention. Face-to-face brief advice in a hospital or clinic was the most frequently used method (87.56%), followed by rehabilitation camps (9.21%).

### Smoking cessation outcomes

At 3 and 6 months, the intention to treat analysis found a continuous smoking abstinence rate (CAR) of 17.49 and 8.33%, respectively. We also found a decrease in 7-day self-reported PAR from 17.49 to 11.22% at 3 and 6 months, respectively. CO-confirmed PAR results, suggest a decrease in CO-confirmed PAR, accounting for 8.18 and 2.2% at 3 and 6 months, respectively.

### Smoking cessation outcomes among patients with CVD or cerebrovascular disease and COPD

Results of smoking cessation rates for patients with CVD or COPD are also summarized in Table 3. At 3 months, intention to treat analysis revealed that 26.36, 26.36, and 5.44% of participants with cardiovascular disease or cerebrovascular disease, respectively, achieved CAR, PAR, and CO-confirmed PAR. By comparison, at 6 months, the smoking cessation outcomes for CAR, PAR, and CO-confirmed PAR were reduced to 13.81, 15.78, and 1.26%, respectively, in the intention to treat analysis.

**Table 3:** Smoking cessation outcomes in overall participants, and smoking cessation outcomes among patients with CVD or cerebrovascular disease, and COPD.

**Outcomes**	**3 Months**	**6 Months**
	***N* (%)**	***N* (%)**
**Intention to treat analysis**	***N*** **= 2,041**	***N*** **= 2,041**
**Per protocol analysis**	***N*** **= 1,495**	***N*** **= 903**
**Continuous smoking abstinence rate (CAR)**		
Intention to treat analysis	357 (17.49)	170 (8.33)
Per protocol analysis	357 (23.88)	170 (18.83)
**Point prevalence abstinence rate (PAR)**		
**7-day self-reported point prevalence**		
Intention to treat analysis	357 (17.49)	229 (11.22)
Per protocol analysis	357 (23.88)	229 (25.36)
**7-day CO-confirmed point prevalence**		
Intention to treat analysis	167 (8.18)	45 (2.20)
Per protocol analysis	167 (11.17)	45 (4.98)
Participants who underwent an exhaled CO test (per protocol analysis)	167/635 (26.30)	45/165 (27.27)
**Cardiovascular disease or cerebrovascular diseases:**		
**Intention to treat analysis**	***N*** **= 239**	***N*** **= 239**
**Per protocol analysis**	***N*** **= 133**	***N*** **= 79**
**Continuous smoking abstinence rate (CAR)**		
CAR (Intention to treat)	63 (26.36)	33 (13.81)
CAR (per protocol)	63 (47.37)	33 (41.77)
**Point prevalence abstinence rate (PAR)**		
7-day self-reported PAR (Intention to treat)	63 (26.36)	37 (15.48)
7-day self-reported PAR (per protocol)	63 (47.37)	37 (46.83)
7-day CO-confirmed PAR (Intention to treat)	13 (5.44)	3 (1.26)
7-day CO-confirmed PAR (per protocol)	13 (9.77)	3 (3.80)
**Chronic obstructive pulmonary disease:**		
**Intention to treat analysis**	***N*** **= 52**	***N*** **= 52**
**Per protocol analysis**	***N*** **= 36**	***N*** **= 22**
**Continuous smoking abstinence rate (CAR)**		
CAR (Intention to treat)	17 (32.69)	9 (17.31)
CAR (per protocol)	17 (47.22)	9 (40.91)
**Point prevalence abstinence rate (PAR)**		
7-day self-reported PAR (Intention to treat)	17 (32.69)	12 (23.08)
7-day self-reported PAR (per protocol)	17 (47.22)	12 (54.54)
7-day CO-confirmed PAR (Intention to treat)	12 (23.08)	4 (7.69)
7-day CO-confirmed PAR (per protocol)	12 (33.33)	4 (18.18)

The effectiveness of the clinics had a downward trend among participants with the chronic obstructive pulmonary disease when compared between 3 and 6 months. At 3 months, intention to treat analysis revealed that CAR, PAR, and CO-confirmed PAR were 32.69, 32.69, and 23.08, respectively. Additionally, at 6 months, the results were 17.31, 23.08, and 7.69, respectively.

## Discussion

Findings from our study demonstrated that the Thai multidisciplinary smoking cessation clinics were effective at assisting individuals in quitting smoking. The 3-month smoking cessation outcomes observed in our study were consistent with earlier research (12). At 3 months, both CAR and PAR were approximately 17.5%. The CAR and PAR dropped at 6 months, at 8.3 and 11.2%, respectively, which was slightly lower than those in prior research (13–20). This present study showed that the CAR and CO-confirmed PAR at 6 months was lower than in other studies (12–16, 20). Previous studies (12, 15, 20) have reported that the CAR and PAR at 3 months ranged between 12 and 66.1%. Additionally, earlier research found that the CAR (17, 19) at 6 months ranged from 10.7 to 28%, and PAR (13, 14, 16, 20) at 6 months ranged between 17 and 66.7%. The same trend was observed when we compared our results with a previous Thai study (9), which found that smoking cessation at 6 months was 31.3% in the intervention group and 13.8% in the control group, and smoking cessation at 6 months with CO-confirmed was 26.6% in the intervention group and 11.3% in the control group.

The COVID-19 pandemic may account for the difference in the CAR and CO-confirmed PAR observed in this study and other studies. The exhaled-CO test was prohibited to prevent viral transmission. Additionally, the pandemic affected clinic follow-up, resulting in an increase in study dropouts (21, 22). Nevertheless, when we estimated CO-confirmed PAR based on a number of participants who underwent an exhaled-CO test, we discovered that the result was comparable to previous research (13, 14, 16, 20) which were 26.3 and 27.3% at 3 and 6 months, respectively. Another possible reason was a difference in study design. Previous studies used randomized controlled trials (RCTs), recruited only participants with specific diseases, and included continuous assistance from a trained family member, whereas this study used a prospective observational design to reflect the real-world implementation and effectiveness of the multidisciplinary team smoking cessation program. Additionally, this current study demonstrated that half of the participants receiving medication received non-standard pharmacological regimens such as special mouthwash or *Vernonia cinerea* (L.) Less (Little ironweed), while other RCTs used nicotine replacement therapy (NRT), bupropion, or varenicline as a standard pharmacological regimen. Thus, the difference between smoking cessation medical regimens may affect the smoking cessation outcomes.

When compared to continued smoking, smoking cessation has been shown to be effective at reducing CVD-related morbidity and mortality (23–27), as well as slowing the accelerated rate of lung function decline and improving survival in patients with COPD (28, 29). The majority of studies in patients with CVD and COPD reported a 6-month or longer continuous abstinence rate. There was considerable variation in the continuous abstinence rate, and we discovered that the results of the present study were comparable to those of previous research (18, 26, 29). However, it should be noted that only 11.9 and 2.56% of participants with cardiovascular or cerebrovascular disease or COPD, respectively, were included in this study.

The effect of a multidisciplinary smoking cessation clinic was evaluated in this study using a prospective observational design that reflects the real-world outcomes of smoking cessation programs, particularly their effectiveness in low- and middle-income countries with limited human resources and budgets. Additionally, this study divided outcomes into self-reported, and biochemical confirmation. Moreover, one of the strengths of the Thai multidisciplinary smoking cessation clinic is the number of clinics that cover all provinces of Thailand. Given Thailand's five-year national NCDs prevention and control plan (2017–2021) to decrease the prevalence of tobacco use in the population, the result of this study could support a decision to add multidisciplinary smoking cessation clinics to part of the official national health program.

Several limitations of the current study should be noted. First, CAR at 1 year is regarded as the gold standard, but we were unable to utilize this result due to the COVID-19 pandemic. In this study, only CAR at 3 and 6 months was reported. The pandemic also affected clinic follow-up; at 6 months, more than half of the participants had not been followed. This important limitation must be considered when interpreting the results of this study. Second, our study was subject to temporal ambiguity and confounding bias due to the observational nature of this study. Nonetheless, we attempted to reduce this type of bias by employing a prospective data collection method. Third, because multidisciplinary smoking cessation clinics are considered standard care for smokers, it was infeasible to include a comparison group of smokers who did not receive smoking cessation services. In addition, each multidisciplinary smoking cessation clinic may offer unique services or activities, depending on their contexts and available resources. This variation could influence the effectiveness of the program. Our study attempted to address this variation by stratified random sampling criteria that took previous setting performance and health regional strata in the sampling method.

## Conclusion

In conclusion, the multidisciplinary team smoking cessation clinics were effective at assisting Thai smokers with or without comorbidities (CVD, cerebrovascular disease, or COPD) in quitting smoking. Our study results support the inclusion of multidisciplinary smoking cessation clinics in the universal health care benefits package. In addition, future research should examine the long-term effects of smoking cessation programs, such as CAR at 1 year, to corroborate the long-term efficacy of smoking cessation programs.

## Data availability statement

The raw data supporting the conclusions of this article will be made available by the authors, without undue reservation.

## Ethics statement

The studies involving human participants were reviewed and approved by Thailand's Ethical Review Committee for Human Subjects Research has approved the protocol for this study, protocol number 24/2562 and document number 51/2019. Written informed consent to participate in this study was provided by the participants' legal guardian/next of kin.

## Author contributions

CC, NC, KT, and SL conceived this paper. CC, NC, and KT developed protocols. CC developed a clinical recruitment strategy. CC developed a data management tool and analysis plan. All authors contributed to the article and approved the submitted version.

## Funding

This work was supported by National Alliance for Tobacco Free Thailand, grant number 61-00-1344-01/2561.

## Conflict of interest

The authors declare that the research was conducted in the absence of any commercial or financial relationships that could be construed as a potential conflict of interest.

## Publisher's note

All claims expressed in this article are solely those of the authors and do not necessarily represent those of their affiliated organizations, or those of the publisher, the editors and the reviewers. Any product that may be evaluated in this article, or claim that may be made by its manufacturer, is not guaranteed or endorsed by the publisher.
